# Using the health belief model to examine adolescent participation in HIV prevention research in Kampala, Uganda: A qualitative study

**DOI:** 10.1371/journal.pgph.0005322

**Published:** 2025-12-01

**Authors:** Zam Nabalwanyi, Rachel Kawuma, Ivy Kayesu, Rwamahe Rutakumwa, Yunia Mayanja

**Affiliations:** 1 Social science department, MRC/UVRI and LSHTM Uganda Research Unit, Entebbe, Uganda; 2 Development Research and Praxis International (U) Ltd., Entebbe, Uganda; 3 Viral Pathogens Epidemiology and Interventions, MRC/UVRI and LSHTM Uganda Research Unit, Entebbe, Uganda; : St John's Medical College, INDIA

## Abstract

Adolescents’ participation in HIV prevention research is essential for developing effective, youth-centered interventions. However, ethical, social, and practical challenges often constrain their involvement. This study explored facilitators and barriers to participation in biomedical HIV prevention research among adolescents aged 14–19 years at risk of acquiring HIV in Kampala, Uganda. As part of the FERDAR study (Feasibility of Enrolling and Retaining Adolescents at Risk of HIV), we conducted a qualitative investigation from June 2019 to June 2020. Participants were purposively selected from among those enrolled in FERDAR. We conducted in-depth interviews with 50 adolescents (30 females, 20 males) and held six focus group discussions with 52 others (27 females, 25 males). Data were analyzed using the framework analysis method, guided by constructs from the Health Belief Model. Key facilitators of participation included increased awareness of HIV risk due to study information, tangible benefits such as transportation reimbursement and access to health services (e.g., cervical cancer screening), and positive interactions with healthcare providers. Conversely, barriers included aversion to study products (e.g., pre-exposure prophylaxis), fear of HIV-positive test results, stigma, and logistical challenges like lack of time or transport funds. At the community level, mistrust and misconceptions about the research and its products further hindered participation. To enhance adolescent involvement in biomedical HIV prevention research, it is vital to address both individual and structural barriers. Promoting access to prevention services and fostering supportive, youth-friendly research environments can improve engagement and ultimately strengthen HIV prevention efforts for this vulnerable population.

## Introduction

Globally, the number of adolescents affected by the human immunodeficiency virus [HIV] remains alarmingly high. In 2024, about 1 million [700,000-1.34 million] older adolescents between the ages of 15 and 19 were living with HIV worldwide. In the same period, 145,000 [37,000–270,000] adolescents of the same age group [15–19 years] were newly infected with HIV. Of those, about 860,000 [550,000-1.1 million] (84%) live in sub-Saharan Africa (SSA) and adolescent girls are infected almost six times more than adolescent boys [1].

Similar to the trends observed in SSA, HIV prevalence in Uganda significantly increased during early adulthood, particularly among women aged 15–19 years and to a lesser extent among men in the same age group in 2022 [2]. These statistics underscore the disproportionate vulnerability of adolescent girls compared to their male counterparts. The increased HIV risk among females compared to their male counterparts has been linked to factors such as early sexual debut, multiple sexual partnerships (often with older men), and engagement in transactional sex [3]. Substance use, including alcohol and drugs, further heightens vulnerability, particularly among males, as it often leads to risky sexual behavior without protection while intoxicated [4,5]. Hence the need to make efforts to reduce these infections.

Currently, there are several biomedical HIV prevention interventions such as condoms and oral pre-exposure prophylaxis (PrEP) that are proven to be effective against HIV when used properly [6]. However, their uptake and adherence among adolescents remain suboptimal due to barriers such as stigma, pill fatigue, limited knowledge, fear of side effects, and sociocultural norms that discourage preventive health-seeking behaviors [7]. Moreover, adolescents’ reluctance to engage in HIV prevention research, including vaccine trials, often stems from safety concerns, misconceptions about clinical studies, and a general mistrust of research processes [8].

Recent advances in HIV prevention technologies offer promising alternatives. Long-acting injectable PrEP agents such as cabotegravir and lenacapavir [9,10] provide extended protection, addressing adherence challenges associated with daily oral PrEP. The dapivirine vaginal ring [11,12] introduces a female-controlled method of prevention, and other innovations including implants, micro array patches, and HIV vaccine are currently in various stages of research and development [13]. While these products have the potential to transform HIV prevention among adolescents, their successful introduction depends on understanding the behavioral, social, and structural factors influencing adolescent participation in biomedical HIV prevention research.

In 2014, the World Health Organization recommended involvement of adolescents in HIV research as a key priority [14]. However, participation remains limited due to ethical dilemmas surrounding the involvement of minors in research, and distrust of the research process [15]. Additionally, such research often requires parental/guardian consent yet adolescents may fear disclosing sensitive information about their sexual behaviors or HIV risk to their caregivers [16]. Further still, socio-economic inequalities, misconceptions about research, and community-level mistrust further complicate efforts to involve adolescents in clinical trials and prevention programs. This creates a complex barrier to recruitment and engagement in HIV prevention studies [15].

Given these realities, it is essential to identify and address the barriers and facilitators influencing adolescent engagement in HIV prevention research. This analysis draws data from the Feasibility of Enrolling and Retaining Adolescents at Risk of HIV Infection (FERDAR) study, which sought to understand adolescent perspectives on biomedical HIV prevention research. Findings from this study aim to inform strategies for enhancing adolescent participation and designing youth-centered interventions that are acceptable, accessible, and effective in reducing HIV incidence among this vulnerable population.

## Theoretical underpinnings

We used the health belief model (HBM) to assess the facilitators and barriers to participation in an HIV prevention research study among adolescents. The HBM examines social behaviour at both individual and community levels. Its underlying principle is that an individual’s perception of risk and the extent to which they perceive the impact of that risk will affect their decision to take action to avoid it [17]. Previous studies have used the HBM to assess prevention interventions among adolescents [16,18]. The model’s six constructs namely, perceived susceptibility, severity, benefits, barriers, cues to action, and self-efficacy help to explain how adolescents assess risk, weigh benefits, and respond to challenges in engaging with prevention studies. Applying the HBM in this study provides a structured approach to examining how adolescents perceive their HIV risk, the benefits and challenges of participating in biomedical prevention research, and the factors that facilitate or hinder engagement. Thus, leading to more acceptability, uptake, and adherence to support successful recruitment and retention in HIV prevention studies.

## Methods and materials

### Participant recruitment

Project field workers identified adolescents from informal settlements in six urban slums of Kampala, namely Kisenyi, Katwe, Namuwongo, Ndeeba, Wandegeya, and Bwaise. They were recruited from sex work locations, bars and lodges and areas where alcohol and drug misuse was common in these slums. Those identified were approached and informed about the services provided at the GHWP (Good Health for Women Project) clinic and invited to come and participate. Snowball approach was also used by encouraging enrolled participants to refer their peers to the clinic for study screening. At the clinic, they were told about the FERDAR study and those who agreed were screened to assess eligibility. Screening into the study was conducted by trained research staff following inclusion criteria such as: HIV negative, willing to undergo quarterly HIV testing and counselling, sexually active in the past 3 months and willing to return to the clinic for follow up visits. A total of 566 were screened and 490 enrolled (297 females, 193 males) into the study. Recruitment, eligibility, and screening into this study have been previously described [19,20].

### Study design and setting

This was a qualitative study nested within the FERDAR adolescent cohort conducted in Kampala, Uganda from March 2019 to December 2020. The aim of the cohort was to assess the feasibility of enrolling and retaining 500 adolescents (200 males and 300 females) aged 14–19 years in a biomedical HIV prevention study.

The adolescent cohort was based at the GHWP clinic, located in Kampala central division, in a place not easily identifiable to the general public, a measure taken to protect the confidentiality and safety of participants [21]. The GHWP clinic was established in 2008 to study epidemiology of HIV and sexually transmitted infections (STIs) among women at risk of HIV infection. As of July 2014, there were over 2,600 female participants and about 140 male partners who were attending the clinic and participating in different research projects [22]. In 2019, the clinic recruited a cohort of adolescent girls and young women (AGYW) at risk of HIV infection, to participate in an oral PrEP implementation research project [23]. Until December 2020 when it was closed, the clinic offered HIV prevention, care, and treatment services such as pre- and post-exposure prophylaxis and risk reduction counselling, sexual reproductive health (SRH) services such as management of STIs and provision of contraceptives to the women, and HIV prevention and treatment to their male regular partners. HIV prevention and SRH services available at the clinic were also given free of charge to FERDAR participants.

### Sample size and data collection

Fifty-two adolescents (27 girls and 25 boys) from the FERDAR study were purposively selected to take part in 6 focus group discussions (FGDs), matched by gender (3 males; 3 females), and categorised in the age groups of 14–15, 16–17 and 18–19 years. A further 50 (30 girls and 20 boys) took part in a single individual in-depth interview each. Maximum variation sampling was used to include participants of varied characteristics like age, gender, work types and locations.

Data were collected over a period of 12 months starting with FGDs as the main source of data from June to November 2019 using a translated FGD topic guide (S1 File). The reason for starting with FGDs was because little was known about the topic of adolescent participation in biomedical HIV research. Later, emerging topics in the FGDs were interrogated through IDIs with the 50 adolescents, from January to June 2020. In both FGDs and IDIs, the facilitators and barriers to participation in biomedical HIV prevention research among young people were explored. All data collection procedures were conducted in private rooms at the clinic by two experienced social scientists proficient in Luganda, which is the local language commonly spoken in the area. Written informed consent was provided to conduct and audio record. The FGDs lasted 40–60 minutes, while the IDIs lasted 30–40 minutes. All participants were reimbursed with approximately ~9 US dollars to compensate for their time and transport costs.

### Data management and analysis

Immediately after data collection, audio files were anonymised using unique study identifiers to protect participants’ identities and ensure confidentiality. Interviews were transcribed and translated from Luganda to English by the same research assistants who conducted them. Audio recordings and written interview notes were stored on password-protected computers and backed up on secure MRC/UVRI and LSHTM Uganda Research Unit servers accessible only to the study team. All consent documents, interview transcripts and fieldnotes were securely kept under lock and key.

**Fig 1:**
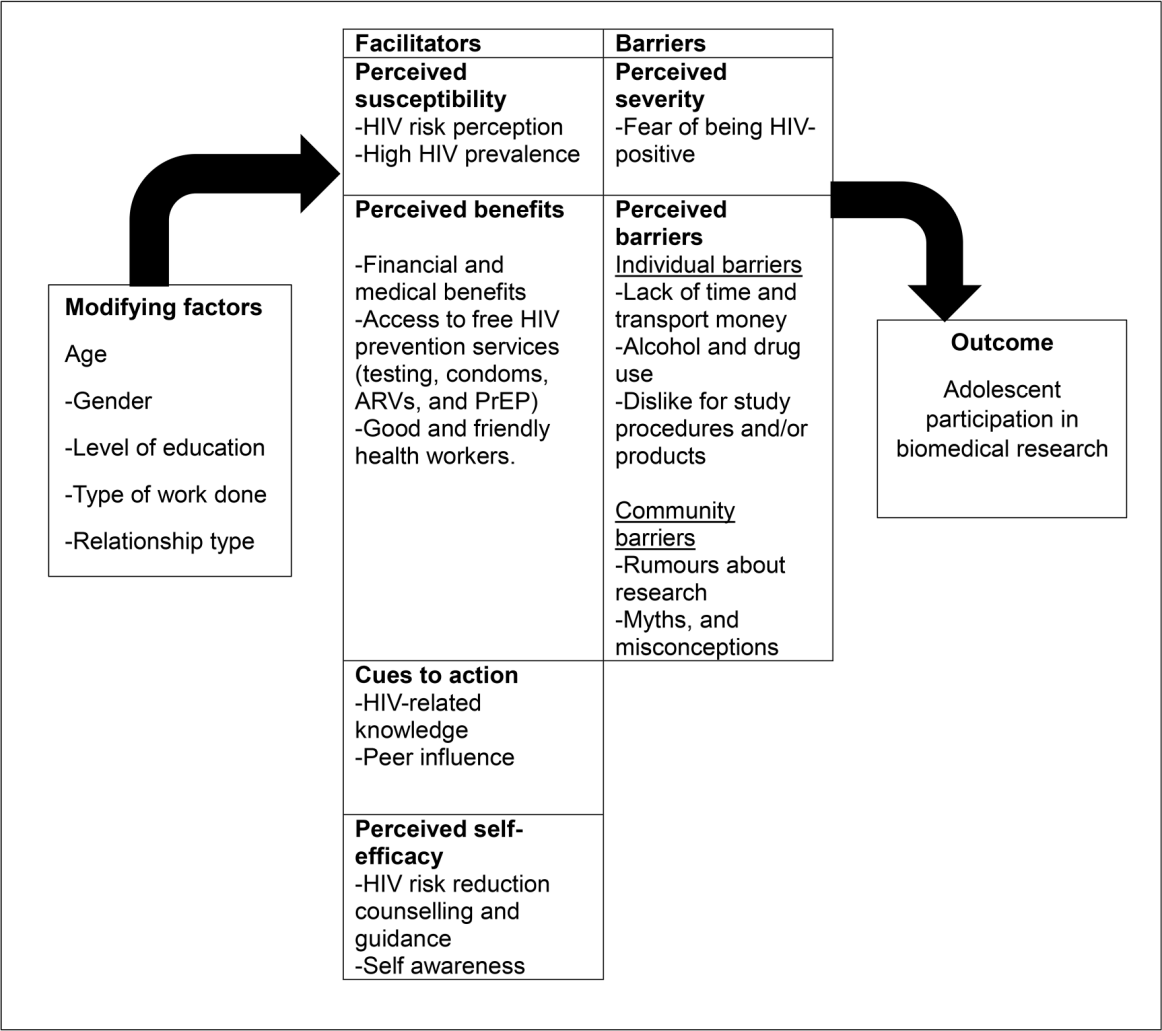
Facilitators and barriers of adolescent research mapped on to HBM.

Data were analysed using a framework analysis approach [24]. Both inductive and deductive coding was used. Two researchers initially read two similar IDI transcripts each to familiarize themselves with the content and to generate inductive codes emerging directly from participants’ accounts. In parallel, deductive coding was used to identify codes related to predefined categories of barriers and facilitators to research participation derived from the interview guide. Identified codes were discussed during a de-briefing meeting with all co-authors and codes with similar or close meanings were grouped together. Thereafter, a coding matrix was developed in excel which guided the systematic coding exercise. All transcripts were coded manually by copying and pasting relevant quotes under specific codes (S2 File). To increase reliability and validity, each transcript was coded twice. The coded data were then organised and charted deductively onto the six theoretical constructs of HBM namely: perceived susceptibility, severity, benefits, barriers, cues to act and self-efficacy which provided a conceptual framework for interpreting (Fig 1).

### Ethics statement

The Uganda Virus Research Institute Research Ethics Committee (GC/127/18/09/658) and the Uganda National Council for Science and Technology (HS 2493) approved the study. All participants were informed about their rights to withdraw from the study at any time and that this would not affect any health care that they were entitled to. All adolescents below the age of 17 years recruited in this study lived independently hence were considered as emancipated and/or mature minors, able to make decisions to participant in HIV prevention research as defined in the National Guidelines for Research Involving Humans as Research Participants [25]. Majority of the participants could read and write, they provided written informed consent and only four were unable to read or write, they indicated consent with a thumb print in the presence of a witness independent of the study.

## Results

### Socio-demographic characteristics

Out of the 50 participants who took part in the in-depth interviews, majority 30 (60%) were between 18–19 years old, 26 (52%) studied up to primary level, 20 (40%) had secondary education while 4 (8%) did not have any formal education. With regards to the type of work done, 37 (74%) described their work as unskilled manual with some of the girls working as waitresses in bars, restaurants and in hair salons while boys served as porters on construction sites, collected scrap metal and used plastic bottles for sale. Finally, most (39, 78%) of the adolescents in the study described themselves as being in temporary relationships with their partners; only 11 (22%), all females identified themselves as “married” because they were in regular relationships and stayed with their partners in the same house. On the other hand, two main characteristics were used to sample FGD participants, namely age and gender, almost equal numbers of females and males were included in the three age categories (Table 1).

**Table 1:** Socio-demographic characteristics of qualitative study participants in the FERDAR study in Kampala, Uganda between June-November 2019.

Participant characteristics	IDI participants n = 50 (%)	FGD participants n = 52 (%)
**Gender**		
Females (F)	30 (60%)	27 (52%)
Males (M)	20 (40%)	25 (48%)
**Age**		
(18–19 years old)	30 (60%) [18F, 12M]	16 (30%) [8F, 8M]
(16–17 years old)	20 (40%) [12F, 8M]	18 (35%) [9F, 9M]
(14–15 years old)		18 (35%) [10F, 8M]
**Education level**		
Did not go to school	4 (8%) [3F,1M]	
Primary education	26 (52%) [13F, 13M]	
Secondary education	20 (40%) [14F, 6M]	
**Occupation**		
Unskilled manual work	37 (74%) [19F, 18M]	
No work	13 (26%) [11F, 1M]	
**Relationship status**		
Regular (married)	11(22%) [only females]	
Not married	39 (78%) [19F 20M]	

In the following section, we describe our findings divided into two themes namely, facilitators and barriers to research participation, mapped on to HBM constructs.

1 **Facilitators to research participation**

This theme explores the factors which enable adolescents to take part in HIV prevention research. These included HIV risk perception, personal motivation, drivers of participation and HIV risk strategies, mapped on to the HBM constructs of perceived susceptibility, perceived benefits, cues to action and self-efficacy in the following sections.

1.1 Perceived susceptibility: HIV risk perception

Two factors predisposed adolescents to HIV risk: having multiple partners and living in high-risk environments. Most of the adolescents recruited in this study were engaged in sexual behaviours that increased their vulnerability to HIV. In both IDIs and FGDs, when participants were asked what influenced their decision to take part in HIV prevention research, susceptibility to HIV infection due to multiple sexual partners featured prominently as reflected in the following quotes:

*“I joined HIV prevention research because I am at risk [of HIV infection] and I have less protection for HIV”. (***Female,19-years old, Primary Education, not married**)

*“I have many girls (sexual partners), that is why I decided to join the HIV prevention research”* (**Male,18 years old, Secondary Education, not married**)

*“When I came here, I thought I had HIV because I slept (have sex) with many girls without using condoms”* **. (Male, FGD, 6-17 age category)**

In addition, adolescents also associated their risk with the fact that they were living in communities with high HIV prevalence thus increasing their likelihood of acquiring HIV. As a result, this motivated them to join HIV prevention research to reduce their vulnerability, as illustrated below:

*“In our area, we have many HIV positive adolescents, and they are promiscuous; I realized that I am at risk, so I had to join (the prevention research).”*(**Female,19 years old, Primary Education, not married**)

“*What influenced my participation in the HIV prevention research is the community we live in; I have sex with multiple girls, so I need to protect myself*”. (**Male,16 years old, Primary Education, not married**)

1.2 Perceived benefits: Personal motivators

These included financial and medical benefits, access to free HIV prevention services (such as testing, condoms, ARVs, and PrEP), and the support and friendliness of health workers.

Participation was associated with tangible incentives, including routine medical care, transport refund for clinic visits, and specialized medical services such as cervical cancer screening, a service typically offered in private settings at a fee. Adolescents cited this as an important motivator for participation in research:

*“Whenever I come to the clinic, I am confident that they are going to give me transport back home. They also give us free treatment when we come to the clinic, so we do not incur expenses to buy medicine”*. (**Female,17 years old, Secondary Education, not married**)

“*The free treatment they give us when we come here facilitates our participation in HIV prevention research. They treat us on every illness; malaria, typhoid to mention but a few*”. (**Male, 19 years old, did not attend school, not married**)

“*The free treatment they give us at the clinic encourages us to participate in HIV prevention research. In some government hospitals, they don’t give us drugs… here, they give us drugs for free and in full doses. In the other hospitals, checking for cervical cancer is very expensive but here it is done for free.*” (**Female FGD, 14-15 age group**)

Another motivator was access to free HIV prevention services such as testing, condoms, and pre-exposure prophylaxis (PrEP):

*“I wanted to get free condoms, and I heard that there is also PrEP so it encouraged me to join because I want to protect myself”. (***Male,19 years old, Secondary Education, not married**)

*“When they find out that you are HIV positive, they give you ARVs and to those that are negative they give us PrEP to protect ourselves against HIV”. (***Female,19 years old, Primary Education, married**)

*“When we come to the clinic, the health workers do HIV tests on us; when they find out that we are negative, they advise us on how to stay negative. They teach us about HIV preventive measures and give us drugs (PrEP pills) that prevent HIV; it encourages us to participate in HIV prevention research”* (**Female FGD,14-15 age group**)

Furthermore, the caring and friendly health workers motivated adolescents to take part in HIV prevention research:

“*The health workers are caring and… when they find out that you are HIV positive, they call you in the private room without your friends noticing and they tell you calmly*” (**Female,18 years old, Secondary Education, not married**)

“*The care they give us when we come to the clinic facilitates our participation in the HIV prevention research. The nurses are very caring and friendly so you can easily confide in them when you have a problem*”. (**Female FGD, 14-15 age group**)

1.3 Cues to action: Drivers to participation

Cues to action refer to the stimuli that trigger individuals to adopt a recommended health behaviour. Among adolescents in this study, key drivers of participation included the desire to acquire HIV-related knowledge and peer influence.

For many participants, limited knowledge about HIV acted as a primary trigger to join the study. This was especially evident among males, who generally reported knowing less about HIV compared to their female counterparts, many of whom had already been part of the adolescent cohort established in 2019. Despite this, both boys and girls acknowledged feeling at risk of infection due to risky sexual practices and alcohol or drug use. In this context, the opportunity to gain knowledge and access HIV services motivated participation. As noted in the quotes below:

“*We also want to know (get knowledge) about HIV, so we are sensitized about it. When we get to know about risky behaviours, we decide to reduce/ stop them because we do not want to get infected.*” (**Male FGD, 16-17 age group**)

“*I wanted to get more knowledge about HIV. I also wanted to know my HIV status so that if I find out that I have HIV, I start ARVs; when I find out that I am negative, I* *continue to protect myself against HIV*”. (**Male, 18 years old, Primary Education, not married**)

“*I wanted to get knowledge about HIV, to know my HIV status. I also wanted to learn how to protect my life and prevent myself from getting infected with HIV*” (**Female,19 years, Secondary level, not married**)

Peer influence was another strong driver of participation. Several adolescents explained that they joined the study after being encouraged by friends and only later realised the benefits. For instance, one recalled that:

“*My friend told me about this clinic, and we came together; she said that they give free treatment and do laboratory tests; that when they find you with any illness, they give you free treatment and refund transport. She encouraged me to come here*”. (**Female, 20 years old, Primary Education, married**)

Similarly, another participant explained that:

“*My friend persuaded me to come here. She came at home and told me, there is a clinic where we go, and they treat us on every illness in case you join the study. She told me many things and is the one who inspired me to come here. Indeed, on the first day I came here, we came together*”. **(Female, 17 years old, Secondary Education, not married**)

1.4 Perceived self-efficacy: HIV risk reduction strategies

Self-efficacy, also referred to as personal control, refers to individual’s perception of having the ability, resources, or opportunities to achieve positive outcomes or avoid negative effects.

self-efficacy was discussed in relation with HIV risk reduction strategies which were enhanced through counselling and guidance provided during participation. These sessions helped adolescents develop self-awareness, evaluate their risk and adopt protective behaviours, thereby improving their self-esteem. For example, one FGD participant explained that she had initially believed she was HIV positive:

“*But after testing and realizing that I am negative, I changed my style of living. The awareness of my status helped me reduce high risk behaviours and start having protected sex*” **(Female FGD, 14-15 age group)**

Similarly, a male participant described how counselling enabled him to modify his behaviour:

“*Before, I used to be a drug addict and was at risk of acquiring HIV but after the counselling, I reduced using drugs [marijuana and alcohol] and now I learnt how to protect myself*”. **(Male FGD, 16–17 age group)**

Overall, adolescents’ participation in HIV prevention research was facilitated by access to free services (HIV testing, condoms, PrEP), medical care, transport reimbursements, and specialized screenings. Counselling and supportive interactions with caring health workers enhanced self-efficacy, enabling participants to assess their risk and adopt protective behaviours. Peer encouragement and the opportunity to gain knowledge about HIV further motivated engagement in the research.

2 **Barriers to research participation**

This emerged as a central theme, reflecting the multiple challenges adolescents face in engaging with biomedical HIV prevention research**.** When asked what might hinder their involvement, participants highlighted negative aspects that could discourage or prevent their continued participation. These were categorized into three sub-themes namely, fear of an HIV- positive result, individual and community barriers, mapped onto the HBM constructs of perceived severity and perceived barriers.

2.1 Perceived severity: fear of an HIV-positive result

Participants’ accounts portrayed HIV as a highly threatening condition with severe physical, psychological, social, and economic consequences. The most frequently reported barrier to participating in biomedical HIV prevention research among both male and female adolescents in this study was the fear of receiving a positive test result. This was expressed through concerns about immediate emotional distress (anxiety waiting for results), long-term life disruption (loss of freedom, depression, hopelessness), physical decline, stigma from peers, treatment burden, and even suicidal thoughts. These perceptions acted as barriers to HIV testing and participation in HIV prevention research. In this regard, adolescents admitted avoiding HIV testing because of the anticipated impact of a positive result on their lives. For instance, one participant explained that:

“*Some adolescents do not want to undergo HIV testing because they are afraid of being told, they are positive. They believe that if they are found positive, they will lose their sense of freedom, their lives will change completely, and they will experience worry and depression*.” (**Female,19 years old, Secondary Education, married**)

“*Some adolescents don’t want to be checked…one said that ‘when I find out that I am HIV positive, I will just take poison and die’. This is because they are afraid and have not received counselling like we did, and lack information about HIV*” (**Male FGD,16-17 age group**)

Similarly, others described the emotional distress associated with waiting for test results:

“*Many adolescents fear testing because they worry, they will be told they are HIV positive. Waiting for results can be stressful, worrying about when they will be called to receive the outcome. This fear discourages their participation in HIV prevention research*.” (**Female FGD, 16-17 age group**).

2.2 Perceived barriers: Individual and community barriers

Perceived barriers which operated at individual level included lack of time, limited money for transport, alcohol and drug use, and dislike of some study procedures or products. At the community level, rumours, myths, and misconceptions about HIV prevention research fuelled mistrust and fear, further discouraging participation as expounded in the following paragraphs.

Time and financial constraints were frequently mentioned as obstacles. Some adolescents explained that competing responsibilities limited their ability to attend clinic visits:

“*What may limit me to participate in HIV prevention research is when I have limited time; by the time they need me, I am very busy, and I can’t make it*”. (**Male,18 years, Secondary Education, not married**)

*“Some of us have limited time to come to the clinic; some of us escape from work and come here to participate in research. You may feel like coming to the clinic to participate in research and you fail to get time. Lack of time affects our participation”*. **(Female FGD, 18-19 age group)**

Others highlighted transport costs as a major barrier:

*“Lack of transport may limit my participation in research. This is because sometimes I may be coming from far and I need a lot of transport so it may limit my participation.”* **(Female 19 years old, Primary Education, not married)**

“*Sometimes we lack transport to come…you may be at home and want to come to the clinic but fail to come because you don’t have transport*.” (**Male FGD, 14-15 age group**)

Substance use was also cited, particularly among boys, who reported that being intoxicated made it difficult to attend clinic appointments:

*“Some of us use drugs [marijuana]; when it is time to come to the clinic, you fail because you are weak, and you cannot tell what is going on. When you take drugs, you lose your senses and forget all about what you are supposed to accomplish in the day”.* **(Male FGD 18-19 age group)**

Another added that:

“*Sometimes we get drunk, and we forget to come*.” (**Male,18 years old, Primary Education, not married**),

Dislike of certain study procedures and products was mostly mentioned by female participants as a hinderance to participation. Cervical cancer screening, for example was viewed as uncomfortable or embarrassing:

“*Some girls fear to be screened for cervical cancer; they feel shy while the health workers are screening them for cancer*” (**Female,19 years old, Secondary Education, not married**)

*“Some girls fear the speculum that they use to check for cervical cancer because they are shy…; they feel uncomfortable while the nurses are checking for cervical cancer”*. (**Female FGD, 14-15 age group)**

Similarly, some adolescents were reluctant to take medication due to pill size, side effects, or fear of long-term treatment:

*“Some adolescents do not want to take drugs…such as the ones taken daily because they are big in size [ARVs]. Others fear pills like the ones they give us for STIs. They don’t feel good when they take these pills; some get nausea, so they fear coming back because they think, they are going to give them more pills”*. **(Female FGD, 16-17 age group)**

Fear of injections was also mentioned, with one participant noting that:

“*Some of us fear injections and nowadays they don’t extract from the finger, they get from the arm, and it is painful.... I want to do frequent checkups, but I fear injections*” (**Female FGD, 14-15 age group**)

In addition to individual-level factors, community-level barriers also hindered adolescents’ participation in HIV prevention research. These barriers largely stemmed from rumours, myths, and misconceptions circulating with in their communities. For example, some adolescents reported hearing that blood collected during research was sold to outsiders, which discouraged participation:

“*Some adolescents say that you take off our blood and sell it to the whites. Peoples’ gossip discourages us from participating in HIV prevention research*” (**Male,19 years old, Primary Education, not married**)

Similarly, other rumours involved fears related to cervical cancer screening:

“*People … say that while screening us for cervical cancer, you remove our uteruses and leave us barren. People do talk a lot, and it discourages me from participating in HIV prevention research*”. (**Female,18 years, Primary Education, not married**)

*“People gossip, they say that the blood you get from us, you sell it to illuminati [cults] and it is that money you give us for transport refund when we come here at the clinic. They also say that while screening us for cervical cancer, you remove our uteruses and hence making us barren. You sell the uteruses to the people who are unable to naturally give birth to children”.* **(Female FGD 18-19 age group)**

Misunderstandings about research procedures also fuelled myths. One participant explained that friends discouraged him from attending the clinic because they believed researchers would collect sexual fluids:

“*When the fieldworker talked to us at recruitment, my friends misunderstood what he said about the research. They said that when we come to the clinic, they are going to tell us to masturbate so that we can give them sexual fluids... and sell them to the whites*” (**Male,16 years old, Primary Education, not married**)

Others feared that cervical cancer screening could affect future fertility:

“*Our neighbour said that I will never get pregnant again because there is a liquid they put on my uterus while checking for cervical cancer that prevents girls from getting pregnant*”. (**Female FGD, 14-15 age group)**

Adolescents’ participation in biomedical HIV prevention research was hindered by fear of an HIV-positive result, which caused anxiety and concern about long-term social, psychological, and physical consequences. Individual-level barriers such as lack of time, transport costs, substance use, and dislike of certain procedures, combined with community-level rumours, myths, and misconceptions, further discouraged engagement. Together, these factors highlight how emotional, practical, and social challenges limit adolescents’ involvement in HIV prevention research.

## Discussion

This study was conducted among male and female adolescents at risk of acquiring HIV, living in urban slums in Kampala, Uganda. We used the health belief model (HBM) to identify what helps or prevents adolescents from taking part in HIV prevention research, to guide youth-centered interventions that are acceptable, accessible, and effective in reducing HIV incidence. Adolescents’ participation in HIV prevention research was influenced by risky behaviors, and limited knowledge, which heightened their perceived HIV risk. While fear of diagnosis posed a barrier, free healthcare, transport refunds, peer influence, and the desire for knowledge encouraged engagement. Counselling and health education enhanced self-efficacy, leading to positive behavior changes such as reduced drug use and safer sex. Our findings align with other HBM-based studies which showed that adolescents’ perceived susceptibility and benefits, such as access to youth-friendly services, motivate participation, while fear of HIV testing and community mistrust remains a key barrier [26,27].

Our findings add to the ongoing discussion about including adolescents in HIV prevention research and emphasize the need to afford them spaces to make independent decisions about participation. Providing them with sufficient information about HIV, which most of them, especially the boys, lacked, increased awareness and helped them take steps to prevent infection. Indeed, this study contributes to evidence that self-efficacy gains from counselling (behavior change such as reduced substance use and safer sex), which provides valuable context about how agency operates differently in this subgroup. Similarly, other studies have shown that understanding one’s risk and the seriousness of HIV can motivate action to prevent it [19,28]. Being aware of risk also improves self-confidence to act, such as testing for HIV and using prevention services. According to the United Nations International Children’s Emergency Fund (UNICEF), ‘adolescents who regularly test for HIV know their status, which is essential for making informed decisions about their health and future’ [1].

This study also showed that peers play an important role in motivating adolescents to join HIV prevention research. Both boys and girls said their friends helped them take part by sharing information and offering social support. Adolescents often trust their peers because they share similar experiences. They are also more likely to do what their friends are doing since they spend most of their time together [29]. Similarly, it is noted that one common reason why adolescents engage in sexual risk behaviors is because they believe their peers are doing the same [30]. Indeed, peer support has been used successfully in other studies to involve young people in health interventions [31,32].

Our results also agree with previous studies showing that perceived benefits motivate people to participate in research [16,33]. In our study, participants valued benefits such as free medical check-ups, cervical cancer screening for young women, STI screening and treatment, transport reimbursements, and regular HIV testing. They also appreciated the friendly staff and being in a youth-friendly space with their peers. Although HIV testing is available in local clinics, participants preferred the privacy and convenience offered in research settings. Other studies among adolescents and young people report that they avoid clinics because of a lack of privacy, long waiting times, negative attitudes of health workers, and discomfort from sharing spaces with adults [34,35]. Knowing what benefits matter most to adolescents can help design youth-friendly services that encourage participation in HIV prevention research.

However, we also found barriers to participation at both individual and community levels. Fear of getting a positive HIV result was highly cited among both males and females as a barrier to taking part in HIV prevention research. Indeed, according to recent statistics, only 29% of adolescent girls and 19% cent of adolescent boys aged 15–19 in Eastern and Southern Africa, the region most affected by HIV have been tested for HIV in the past 12 months and received the result of the last test [1]. Like in other studies [36–38], fear of HIV testing is linked to stigma, the fear of being labeled as HIV positive and worry about coping with a positive result. At the community level, there were rumors about research procedures, likely due to lack of knowledge about the study. Such rumors heighten perceived barriers by reinforcing mistrust, fear, and stigma, which can override individual perceptions of susceptibility and benefits, leading adolescents to avoid participation despite recognizing personal risk or potential advantages. Addressing such barriers during research can help increase engagement and improve retention in prevention studies as documented elsewhere [39]. To address this, studies can use community engagement approaches to provide clear and accurate information about research to community members [40].

Finally, gender differences were minimal in terms of what motivated participation. However, there were some salient gender specific barriers that shaped adolescents’ participation in HIV prevention research and have important implications for study design. For instance, female participants highlighted discomfort with procedures such as cervical cancer screening and expressed anxiety over community rumors linking such procedures to infertility. On the other hand, male participants described alcohol use and drug consumption to be interfering with their ability to attend visits or adhere to study requirements. Similar findings have been reported in earlier studies [4,41], Thus the importance of gender-sensitive interventions that address the specific risks faced by adolescent boys and girls. For girls, the interventions will aim at creating private, supportive environments with tailored counselling to demystify reproductive health procedures and incorporating flexible scheduling and linkage to substance use support services for the boys. Addressing these gendered challenges can improve recruitment, retention, and the overall inclusivity of biomedical HIV prevention research among adolescents.

## Strength and limitations

The strength of this study lies in its methodology. Including a diverse range of young people (across genders and age groups) in both interviews and focus group discussions enabled us to gain insights into their experiences as they participated in the study.

In addition, using the Health Belief Model (HBM) allowed us to explore the factors that young people in this study considered important when making decisions about participating in HIV prevention research. We observed that increasing awareness of their HIV risk equipped them with knowledge, which helped them make decisions whether to participate and, in turn, contributed to their self-efficacy. Furthermore, we identified an intersection between individual and community-level factors that acted as barriers to participation in HIV research.

However, there were some limitations noted. For instance, we interviewed emancipated and/or mature minors who did not require parental consent to participate in the study because they were considered independent or living as adults. Their experiences may differ from those of adolescents under the care of parents or guardians, who may lack the autonomy to make decisions about participation or to discuss personal experiences. However, the study highlights real dilemmas faced by adolescents at risk of acquiring HIV who may not have access to HIV prevention programs.

Secondly, the benefits received as part of participating in this study such as routine medical care and transportation reimbursement could have contributed to their motivation to take part and created a perceived obligation to participate. This may not reflect the actual willingness to engage in routine HIV prevention services offered at health facilities that lack incentives, experience drug stock-outs, and face shortages of healthcare staff.

## Conclusion

We explored the facilitators and barriers to participation in biomedical HIV prevention research among adolescents and young people aged 14–19 years. The study found that increasing knowledge and awareness about HIV in this population contributed not only to their acceptance of joining the study but also to their continued engagement. While the benefits provided motivated participation, challenges remained at both individual and community levels. Addressing these barriers through the provision of more knowledge and collaboration with community stakeholders could improve young people’s participation in biomedical research. This, in turn, could lead to greater acceptability, uptake, and adherence to HIV prevention interventions, as well as successful recruitment and retention in prevention studies.

More studies are needed to examine how facilitators and barriers for adolescent participation in research evolve over time and how interventions can sustain engagement beyond initial enrolment. Further, community-engagement research could help address persistent rumors and stigma, while comparative studies across gender and socio-economic groups may highlight tailored strategies for boys and girls. Finally, innovative approaches such as digital platforms and harm-reduction strategies for substance use should be investigated to improve participation, adherence, and overall impact of HIV prevention interventions among adolescents.

## Supporting information

S1 File Qualitative FGD discussion topic guide. (DOCX)

S2 File Thematic analysis framework. (DOCX)
