# Supplementary material for: Using the health belief model to examine adolescent participation in HIV prevention research in Kampala, Uganda: A qualitative study
Source: PLOS Glob Public Health. 2025 Dec 1;5(12):e0005322. doi: 10.1371/journal.pgph.0005322 (PMC12668540; doi:10.1371/journal.pgph.0005322)
Supplement: S1 File — (DOCX) [file pgph.0005322.s001.docx]

**FGD GUIDE**

**Purpose of FGD:** Get an understanding of the facilitators and barriers to adolescent participation in HIV prevention research and their perceptions and attitudes towards on-demand PrEP as an HIV prevention method.

Date: ……… Start time: ………… End Time……

Venue of FGD…………..

Facilitator’s names: ………… Note takers initials: ……………..

**Administer informed consent.** *(Read out loud the information on the form to the participants*)

***I introduce them to the recorder and ask them whether they are all okay with recording the discussion.***

| “Thank you for agreeing to take part in this discussion. As I mentioned this group discussion is part of the study that you are taking part in. Some things we talk about today are not yet available and may become available soon (on- demand PrEP), but your opinion is still important to us. There is no judgement on anyone even though we will be talking about sensitive issues like sexual behaviour. All opinions are welcome, and there are no right and wrong answers; only differing points of view. We ask you not to share anything said during this group discussion outside the discussion. Your honest discussion will help us learn the best ways to involve young people in research, learn about their health problems and work with them to find out how best to help them take up health interventions that are beneficial to them. The information we get from you will also help us to design appropriate education messages and plan how best to work with adolescents in future HIV prevention research. The discussion will last approximately **1:30 minutes”** |
| --- |

1. **Knowledge about HIV**

- Knowledge on HIV Risk behaviours (transmission, adolescents more at risk (age, sex)

**Byemumanyi ku katyabaga k’okukwatibwa akawuka akaleeta mukenenya (Ensasanya, abavubuka abasinga okuba mu katyabaga k’okukwatibwa akawuka akaleeta mukenenya [ekikula, emyaka])**

1. **HIV preventive measures**
   - Knowledge on HIV prevention measures (most commonly used measures)

**Byemumanyi ku ngeri yokuziyiza akawuka akaleeta mukenenya (Ziriwa zemusinga okukozesa)**

- - Sources of information on HIV preventive measures

**Amawulire agakwata kungeri yokuziyiza akawuka akaleeta mukenenya mugajawa.**

- - Motivators of adolescent participation in HIV prevention research

**Ebisobozesa abavubuka okwetaba mu kunonyereza kwo kuziyiza akawuka akaleeta mukenenya.**

- - Barriers to adolescents’ participation in HIV prevention research

**Ebiremesa abavubuka okwetaba mu kunonyereza kwo kuziyiza akawuka akaleeta mukenenya.**

**3. Perceptions about on-demand PrEP.**

- Knowledge about PrEP (sources of information on PrEP, accessibility of PrEP)

**Byemumanyi ku PrEP (amawulire agakwata ku PrEP mugajawa, n’engeri yokufuna mu PrEP)**

- Perceptions on taking on- demand PrEP to prevent HIV (other people’s views on PrEP, family, friends and community)

**Endowooza ku kumira PrEP ng’amuli mubwetaavu (nga tonaba kwegatta okusobola okuziyiza akawuka akaleeta mukenenya *(endowooza zabantu abalala ku PrEP, ab’enganda, emikwano n’ ab’ekitundu)***

*-PrEP effectiveness*

***Omugaaso gwa PrEP***

*-Side effects of PrEP*

***Obuzibu obuyinza okuva ku kumira PrEP.***

- Perspectives on time for taking PrEP (days, months, years, lifetime)

***Endowooza ezekuusa kubbanga lyokumiriramu PrEP (ennaku, wiiki, emyaka, luberera)***

1. *Current situation (how they recommend to take it)*

***Embeera eriwo kati (Engeri gyebabagamba mu okulimira)***

*Ii) Preferred time for taking PrEP (why)*

***Ebbanga lye mwandiyagalidde mu okumiriramu PrEP (lwaki)***

- Perspectives on how adolescent’s knowledge about PrEP can be improved.

***Endowooza ezekuusa ku ngeri abavubuka gyebayinza okweyongera okumanya***

***ebikwata ku PrEP.***

- Attitudes towards on- demand PrEP as an HIV prevention method *(adolescents, family and community)*

**Endowooza ku kumira PrEP ng’oli mubwetavu (nga tonetaba mubikolwa eby’okwegatta nga enkola ey’okuziyiza akawuka akaleeta sirimu *(abavubuka, abenganda n’abekitundu)***

- Influence of social networks on PrEP *(friends, family, community)*

**Emikwano, abenganda n’abekintundu bawagira batya oba banafuya batya enkozesa ya PrEP**

- Influence of PrEP on other (HIV) preventive measures. *( like condoms, abstinence)*

**Enkola eno eya PrEP etumbula atya oba enafuya etya enkola endala/ emitendera emirala mu kuziyiza akawuka akaleeta mukenenya.**
