# Supplementary material for: Using the health belief model to examine adolescent participation in HIV prevention research in Kampala, Uganda: A qualitative study
Source: PLOS Glob Public Health. 2025 Dec 1;5(12):e0005322. doi: 10.1371/journal.pgph.0005322 (PMC12668540; doi:10.1371/journal.pgph.0005322)
Supplement: S2 File — (DOCX) [file pgph.0005322.s002.docx]

|  | |  |  |  |
| --- | --- | --- | --- | --- |
| **Thematic analysis table: facilitators and barriers to adolescent research participation mapped on to HBM constructs** | | | | |
| **Theme 1** | **Sub-themes** | **Codes** | **excerpts** | **HBM Construct** |
| **Facilitators of research participation** | HIV risk perception | Multiple sexual perceptions | *I joined HIV prevention research because I am at risk [of HIV infection] and I have less protection for HIV. I wanted to know how best I can protect myself from acquiring HIV*. (Female 19 years) *I have many girls (sexual partners) that is why I decided to join the HIV prevention research* (Male 18 years)  *When I came here, I thought I had HIV because I slept (have sex) with many girls without using condoms.* (FGD males 16-17 years) | Perceived susceptibility |
|  |  | High risk environments | *In our area, we have many HIV positive adolescents and promiscuous; I realized that I am at risk, so I had to join*. (Female 19 years)  *What influenced my participation in the HIV prevention research is the community we live in; I have sex with multiple girls so I need to protect myself*. (Male 16 years) |  |
|  | Personal motivation | Financial and medical benefits | *When I learnt that they give us transport refund when we come to the clinic, it encouraged me to participate in the study. Whenever I come to the clinic, I am confident that they are going to give me transport back home. They also give us free treatment when we come to the clinic, so we do not incur expenses to buy medicine*. (Female 17 years)  *The transport they give us after giving us treatment. Sometimes we leave our work and come to the clinic, but we end up not losing because they give us transport. Sometimes we are better of the person who went to work, when you go to work, you may end up not getting profits that you get when you come here at the clinic*. (Female 19 years) *It inspires us to come and join HIV prevention research. The free treatment they give us when we come here facilitates our participation in HIV prevention research. They treat us on every illness; malaria, typhoid to mention but a few.* (Male 19 years)  *The free treatment they give us at the clinic encourages us to participate in HIV prevention research. In some government hospitals, they don’t give us drugs… here, they give us drugs for free and in full doses. In the other hospitals, checking for cervical cancer is very expensive but here it is done for free.”* (FGD, females 14-15 years)  *To be honest to you, if there was no transport, none of us would be here. We would rather spend time making money than coming here. However, when we come here, they give us money for the time and transport back home which inspires us to come* (FGD female 18-19 years) | Perceived benefits |
|  |  | Access to free HIV prevention services (testing, condoms, ARVs,PrEP) | *when they find out that you are HIV positive, they give you ARVs and to those that are negative they give us PrEP to prevent ourselves from HIV.* (Female 19 years)  I also *wanted to know my HIV status in that when I find out whether I am infected, I start ARVs; when I find out that I am negative, I continue to protect myself against HIV*. (Male 18 years)  *I wanted to get free condoms, and I heard that there is also PrEP so it encouraged me to join because I want to protect myself.* (Male 19 years) *I wanted to find out whether I have HIV or not. I also wanted to know how to prevent myself from HIV. I also wanted to know whether I have hepatitis B or not. When they screened me for hepatitis B, they said my liver is fine and I have protection, so I was not vaccinated for hepatitis B* (Male 19 years)  *Presence of free HIV testing services, wanting to know my Current status and getting knowledge on how to prevent myself from HIV facilitates my participation in HIV prevention research* (FGD,Girls 14-15 years) *Wanting to get free condoms facilitates our participation in HIV prevention research* (FGD,Boys 18-19 years)  *We get to learn HIV preventive measures, getting to know about new preventive measures like use of PrEP and others. When I come here and they teach me about HIV; I can’t risk playing sex with anyone, without using protection* (FGD,Boys 18-19 years). |  |
|  |  | Good attitude of health workers | *“The health workers are caring and… when they find out that you are HIV positive, they call you in the private room without your friends noticing and they tell you calmly”* (Female 19 years)  *the health workers in other health centers mind their own business; they don’t care. But here the nurses are friendly, they care for us; when I’m sick, I don’t mind putting in transport to come here for treatment and leave the other hospitals around home. This is because of the care they provide to us* (FGD Girls 18-19 years) The care they give us when we come to the clinic facilitates our participation in the HIV prevention research. The nurses are very caring and friendly so you can easily confide in them when you have a problem. (FGD Girls 14-15 years) |  |
|  | Drivers to participation | Knowledge about HIV/ prevention measures | *I wanted to get knowledge about HIV. I also wanted to know my HIV status; getting to know what I should take or leave. I wanted to learn how to improve my life and how to prevent myself from HIV* (Female 19 years**)** *“I wanted to get more knowledge about HIV. I also wanted to know my HIV status so that if I find out that I have HIV, I start ARVs; when I find out that I am negative, I continue to protect myself against HIV”. (Male 19 years)*  *Being at risk, I wanted to know how to prevent myself from HIV****.*** *(Female 18 years)*  *We also want to know (get knowledge) about HIV so that we don’t fall in the trap unknowingly; when we get to know about risky behaviours, we decide to reduce/ stop them because we don’t want to get infected (FGD Boys* *16-17 years****)*** | Cues to action |
|  |  | Peer influence | *My friend persuaded me to come here. She came at home and told me, there is a clinic where we go and they treat us on every illness in case you join the study. She told many things and she is the one who inspired me to come here* (Female 17 years) *My friend told me about this clinic and we came together; she said that “at the Good Health for Women Project clinic, they give free treatment; they do laboratory services, when they find you with any illness they give you free treatment and also refund transport. She encouraged me to come here; when I came (here) they checked me and found out that I have wounds in my private parts, they gave me treatment and candida cleared.* (Female 20 years)  my friends influenced me to come to the clinic. They told me that when you come to the clinic (Male 18 years) |  |
|  | HIV risk reduction | Self awareness | *The counseling I got from health workers facilitated my participation in HIV prevention research. Before I used to be a drug addict and I was at risk of HIV. Having received counseling, I reduced on the drugs (marijuana and alcohol) and now I learnt how to manage myself* (FGD Boys 16-17 years old)  *I came here (to the clinic) believing that I am HIV positive but after testing and realizing that I am negative, I changed my style of living. The awareness of my status helped me reduce high risk behaviours and start having protected sex* (FGD Girls 14-15 years old)  *Some of us, by this time we would be drunk already but at least we have participated in something productive* (FGD Boys 16-17 years old) *Whenever you are about to involve in risk behaviors, you remember what the nurse told you and you decide to keep calm. Wherever I am going to have sex with the person I am not sure of her status, I remember what I was told at the clinic; I ran to pick a condom so that I prevent myself from HIV* (FGD Boys16-17 years old) | Perceived self-efficacy |
| **Theme 2** | **Sub-themes** | **Codes** | **Excerpts** | **HBM Construct** |
| **Barriers to research participation** | Fear of HIV positive result | Consequences of being HIV positive | *Telling someone that he/she is HIV positive discourages adolescents to participate in HIV prevention research. Many adolescents fear to be checked (tested) because they think that they will be told that they are HIV positive. When you are waiting for results, you can be on tension, even the adults fear to do an HIV test. You can be like “when are they calling me to receive the results?” You can be scared. This discourages adolescents’ participation in HIV prevention research*. (FGD Girls16-17 years old) *Some adolescents don’t want to be checked for HIV. They fear to be checked for HIV; they don’t want to be told that they are positive. Some think that when they find out that they are positive, they will no longer be free; their life will never be the same. They will get worried and become depressed.* (Female 19 years)  *Some fear their friends to get to know that they are HIV positive; initially they may have checked for HIV and they find out that they are infected so they are scared of their friends finding out that they are HIV infected* (Male 16 years**)** *Some adolescents don’t want to be checked; he can say that “when they find out that I am HIV positive, I will just take poison and I die.” This is because they have fear, they have not received counselling that we got; they are ignorant about HIV information* **(**FGD,Boys16-17 years**)** | Perceived severity |
|  | Individual barriers | Lack of time and transport money | *Lack of money for transport may limit my participation in HIV prevention research. This is because sometimes I may be coming from far and it needs much transport so it* (Female 19 years)  *What may limit me to participate in HIV prevention research is when I have limited time; by the time they need me, I am very busy and I can’t make it* (Male 18 years) *Some of us have limited time to come to the clinic; some of us escape from work and come here to participate in HIV prevention research. You may feel like coming to the clinic to participate in HIV prevention research and you fail to get time. Lack of time affects our participation in HIV prevention research* (FGD, Girls14-15 years) *Sometimes we lack transport to come for HIV prevention research. You may be at home and wanting to come to the clinic; you fail to come because you don’t have transport* (FGD,Boys 14-15) | Perceived barriers |
|  |  | Alcohol and drug use | *Sometimes we get drunk and we forget to come* (Male 18 years), *May be when I am drunk, when I don’t have strength to come* (Male 19 years) *Some of us use much drugs (marijuana); when you reach time to come to the clinic, you fail because you are weak and you can’t tell what is going on*. (FGD, Boys16-17 years)  *Use of drugs and alcohol (all giggled); you may wakeup with hangover, and you fail to come to the clinic. When you take drugs, you lose your senses and fail to come to participate in HIV prevention research* (FGD, Boys14-15) |  |
|  |  | Dislike for study procedures and/or products | *Some girls fear to be screened for cervical cancer; they feel shy while the health workers are screening them for cancer* (Female 19 years) *Others fear injections and don’t want to take off their blood* (Female 17 years) *Some girls fear the speculum that they use to check for cervical cancer because they are shy; when you tell your friend to come to the clinic so that they check her for cervical cancer, she will tell you “I am not coming because she feels shy”. She doesn’t want to be seen by the nurse. They feel weird while the nurses are checking for cervical cancer*. (FGD,Girls14-15) *Some of us fear injections and nowadays they don’t extract from the finger, they get from the arm and it is painful. Whenever it gets to 3 months when I have to do checkup, I get worried because I fear injections. I want to do frequent checkups, but I fear injections* (FGD,Girls14-15 years) |  |
|  | Community barriers | Rumours about research | *Some people were saying that when you come here (at the clinic), they take off your blood and sell it to whites. Initially, they discouraged me* (Male 18 years) *People in my community say a lot of things... that you [researchers] are just testing drugs on us to see whether they work, otherwise you would not be concerned about us without a motive* (Female 17 years)  *People gossip, they say that you take off our blood and sell it to the whites so that you can get money. They say that while screening for cancer you take our uteruses leaving us barren...and it discourages me from participating in HIV prevention research*. (Female 18 years)  *Some people say that “health workers take our blood to witch doctors” so that discourages us from coming to the clinic* (FGD, Girls18-19 years) *Some people say that “the blood you get from us, you sell it to illuminati and it’s the money you refund to us when we come here at the clinic.” They also say that “while checking for cervical cancer, you remove our uteruses hence making us barren.” You sell the uteruses to the people who don’t produce*. (FGD, Girls18-19 years) |  |
|  |  | Myths and misconceptions | *Some friends discouraged us from coming to the clinic because they said that “they are going to take our sexual fluids and sell them to the whites, yet they had never come here [clinic] before* (Male 16 years)  *They said that “there are certain liquids they (health workers) put on the uterus that make us (girls) barren.” Our neighbour said that “I will never get pregnant again because there is a liquid they put on my uterus while checking for cervical cancer that prevents girls from getting pregnant*. (FGD, Girls14-15 years) |  |
